# The ectopic vesical pheochromocytoma a diagnostic and therapeutic challenge case report and literature review

**DOI:** 10.1016/j.ijscr.2020.11.132

**Published:** 2020-12-02

**Authors:** Issam Jandou, Amine Moataz, Mohammed Hicham Mouqtassid, Mohammed Dakir, Adil Debbagh, Rachid Aboutaieb

**Affiliations:** a University Hospital Center Ibn Rochd Casablanca, Morocco; b Faculté de médicine et de pharmacie Casablanca, Morocco

**Keywords:** Ectopic pheochromocytoma, Bladder pheochromocytoma, Methoxylated derivative, Partial cystectomy

## Abstract

**Introduction:**

Extra-adrenal localization of pheochromocytoma is rare. Its clinical revelation is paroxysmal but maybe in the form of permanent hypertension resistant to treatment. The main problem with these tumors is to affirm their benignity or malignancy, ectopic pheochromocytomas have a malignant development once in two.

**Case presentation:**

We report the observation of a 39-year-old woman hypertensive on Amlodipine. She presents for sweating, headache, and palpation. The dosage of urinary metabolites revealed a marked increase in metanephrine and normetanephrine. Imagery had shown, at the expense of the left lateral wall of the bladder, a budding tissue formation with irregular contours which was significantly enhanced after injection of PDC. The diagnosis of an ectopic pheochromocytoma was accepted. The patient underwent a partial cystectomy, the postoperative consequences of which were unremarkable.

**Discussion:**

Threatening secretory syndromes are rare medical situations that require urgent management. Pheochromocytoma can be seen at any age from childhood to old age. The treatment of pheochromocytoma is surgical, once the stages of positive and topographic diagnosis have been carried out, the patient must be immediately entrusted to a team of trained surgeons and anesthetists. The prognosis remains unpredictable, hence the interest in prolonged monitoring.

**Conclusion:**

Pheochromocytoma is a tumor developed at the expense of chromaffin tissues, extra-adrenal localizations exist, but remain rare. It is a pathology that presents a diagnostic and therapeutic dilemma in which patients require regular and prolonged postoperative monitoring to detect a possible metastasis or recurrence.

## Highlights

• Pheochromocytoma is a tumor developed at the expense of chromaffin tissues.
• Extra-adrenal localization of pheochromocytoma is rare.
• The main problem with these tumors is to affirm their benignity or malignancy.
• Ectopic pheochromocytomas have a malignant development once in two.
• Ectopic pheochromocytoma presents a diagnostic and therapeutic dilemma.

## 1 Introduction

The extra-adrenal pheochromocytoma or paraganglioma is a tumor of neuroectodermal origin growing at the expense of chromaffin tissue. The adrenal site as usual (90%), the extra-adrenal location is rare representing 10% of pheochromocytomas. This can be located in the sympathetic and parasympathetic vertebral nodes. ZuckerKandel's organ involvement is by far the most common ectopic location [1].

Its classic clinical expression is paroxysmal (sweating, headache, tachycardia) associated with hypertension (hypertension). These elements may be missing and the condition may take on the mask of permanent treatment-resistant hypertension.

Its treatment is surgical. The course is marked by the risk of late recurrence even when the tumor appeared to be benign.

We report an observation of bladder pheochromocytoma by studying the clinical aspects, the contribution of imaging in the diagnosis of extra-adrenal pheochromocytoma, and establishing a strategy for the therapeutic management of these tumors. This work was reported in accordance with the 2018 SCARE criteria [2].

## 2 Case presentation

Ms. H.N aged 39, multiparous, without profession followed for hypertension for more than a year on calcium channel blocker: amlodipine 10 mg/d. The onset of symptoms was more than a year before admission by the onset of hypertensive paroxysmal attacks, triggered by emotion, a headache of moderate-intensity, unspecified topography, palpitations, and generalized sweating with redness of the face, without hematuria or other urinary signs or notion of permictional symptoms. The whole has evolved in the context of apyrexia and unencrypted weight gain.

The clinical examination on admission found a patient in good general condition, weight = 60 kg, height = 1.52 m, body mass index (BMI) = 25 kg/m^2^, conjunctiva, color, eupneic, apyretic at 37° 2. The cardiovascular examination noted a paroxysmal hypertension at 220 mmHg for the systolic and 140 mmHg for the diastolic, the pulse was at 100 beats/min. There was no palpable mass, nor lumbar contact, the hepatic arrow was at 11 cm, the spleen was not palpable.

The ionogram revealed a serum at 140 meq/l, the serum potassium at 5 meq/l, the chlorine at 110 meq/l. the blood urea level was 0.29 g/l, the serum creatinine was 6.3 mg/l. The blood sugar was raised to 2.4 g/l. hemoglobin at 12.9 g/100 mL, Platelets at 507,000/mm^3^, white blood cells at 5,200/mm^3^. SV was accelerated to 30 mm in the first hour. The dosage of urinary metabolites, metanephrines 0.40 mg/24 h (0.04 to 0.20), normetanephrines 8.41 mg/24 h (0.07 to 0.38).

**Fig. 1:**
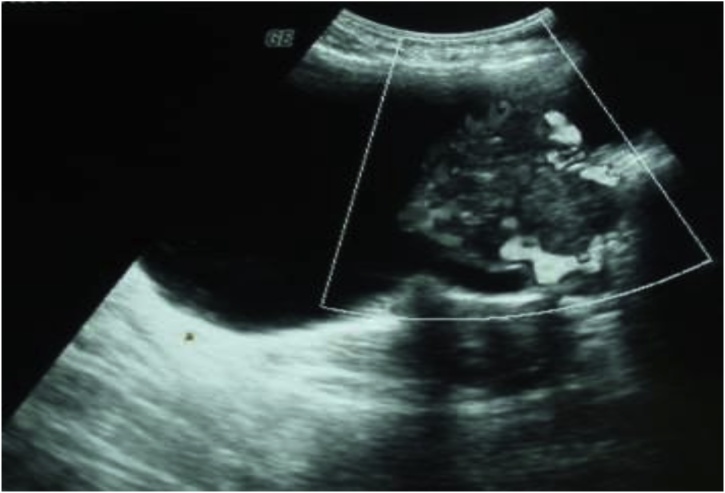
Ultrasound appearance of the bladder mass.

**Fig. 2:**
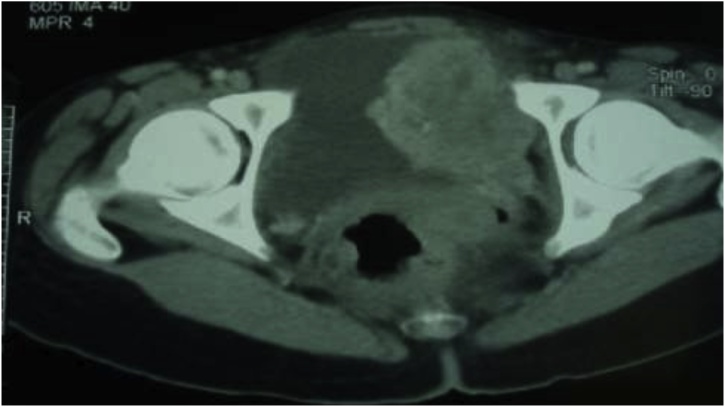
CT of the bladder mass.

Ultrasound and abdominal CT, They had highlighted, at the expense of the left side wall of the bladder, a budding tissue formation with irregular contours which was significantly enhanced after injection of PDC, measuring 53 × 50 × 60 mm. This mass is the seat of small calcifications. The adrenals were intact. No intra or retro-peritoneal lymphadenopathy, normal-looking liver (Fig. 1, Fig. 2).

Meta-iodo-benzyl-guanidine scintigraphy labeled with iodine-131 was not done. C- the chest x-ray did not reveal any anomalies. A check-up for multiple endocrine neoplasia was done and was negative (normal cervical ultrasound, normal parathyroid hormone, and calcitonin measurements). The diagnosis of vesical pheochromocytoma has been strongly suspected, hence the need for surgical exploration.

The patient had received antihypertensive treatment based on Amlodipine 10 mg 1cp/d, Doxazosine 2 mg 1cp/d, Atenolol 100 mg 1cp/d.

The operation took place in 2 stages:

First step: cystoscopy, the exploration of which has found a thickening of the left lateral wall near the bladder neck, with no visible budding mass in the endovesical.

Second step: Tumorectomy, Partial cystectomy passing 2 cm from the tumor. During the manipulation of the bladder, the patient presented well-controlled hypertensive peaks at 220/160 mmHg.

**Fig. 3:**
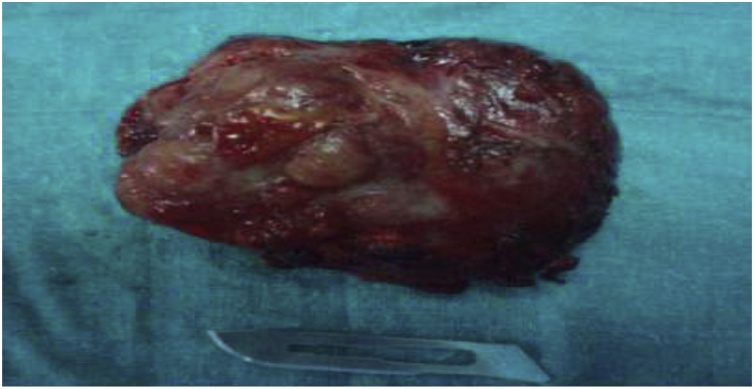
Bladder mass.

Macroscopic anatomopathological examination was a well-encapsulated, firm tumor weighing 40 g and measuring 6.5 cm from the major axis (Fig. 3). When cut, it was yellow-chamois, not very hemorrhagic, and not very necrotic. The histological examination brought the diagnosis of certainty of a chromaffin tumor by highlighting cytoplasmic granulations after staining with chromate-potassium dichromate.

The postoperative follow-ups were simple with normalization of the blood pressure on day 1 of the postoperative (100/70 mmHg), the pulse was at 77 beats/min. The dosage of metanephrine and urinary normetanephrine at 1 week postoperative was found to be normal.

The clinical, ultrasound, and biological follow-up at 3 months and 6 months was without abnormality.

## 3 Discussion

Threatening secretory syndromes are rare medical situations that require urgent management [1]. Pheochromocytoma is a rare tumor, the cause of which is unknown, its prevalence is 1 in 10,000 [3], it is described as being responsible for less than 1% of arterial hypertension [4]. Extra-adrenal localization is rare, it is more common in children than adults [5], and it represents 10–15% of pheochromocytomas. The first case was described by Zimmerman in 1953, it was a pheochromocytoma with a bladder localization. The ectopic localizations are in 13% vesical. Localization of the Zuckerkandl organ is the most common ectopia. In 1976 Glenn reported 50 cases of pheochromocytoma of the Zuckerkandl organ.

Pheochromocytoma can be seen at any age from childhood to old age. The average age of predilection is 40 years with extremes ranging from 10 to 74 years [6]. Exceptional observations have been reported in newborns and beyond 80 years of age.

The majority of pheochromocytomas are symptomatic. The symptoms being linked to the secretion of catecholamines (vasomotor disorders, arterial hypertension, diabetes, weight loss) or tumor extension in the case of malignant pheochromocytomas. The others are accidentally discovered during a radiological examination or screening for familial forms [7]. Others are discovered at autopsy in the Mayo Clinic's autopsy series [8].

The classic clinical expression of pheochromocytoma is the triad: headache, sweating, and palpitations in a hypertensive patient. However, it can take various clinical forms depending on the site, nature, and tumor volume. Sometimes it is responsible for more unusual cardiovascular manifestations such as acute edema of the lung, myocardial involvement, or acute symptoms.

Urinary manifestations, namely The per or post-voiding occurrence of general malaise (hypersudation, palpitation, or headache) which accompanies a hypertensive flare is very suggestive of pheochromocytoma with bladder localization [9]. These signs are due to the release of catecholamines, with a full bladder, during and immediately after urination. Boccon-Gibbod et al. had emphasized that post-voiding angina pectoris should cause a vesical localization of a pheochromocytoma to be sought.

Hematuria is also a warning sign and may be accompanied by kidney failure. Pollakiuria and dysuria are sometimes reported [8].

Recent progress, made in the knowledge of the genetic mechanisms responsible for pheochromocytomas, reveals the role of germline mutations of different genes involved and allows to improve the diagnosis and the family care. The rule that 10% of pheochromocytomas are bilateral, 10% extra-adrenal, 10% familial, and 10% malignant is questioned today. Although most pheochromocytomas are sporadic, genetic studies in recent years have shown that 25–30% of them are hereditary, due to a germline mutation in the RET, VHL, NF1 genes or genes encoding the subunits B, D and C of succinate dehydrogenase from the Krebs cycle [10].

The basis for the diagnosis of pheochromocytoma is the demonstration of excessively high secretion and consequently elimination of catecholamines or their metabolites. These compounds however have different diagnostic performances.

The dosage of adrenaline and urinary free norepinephrine is less reliable than that of their methoxylated metabolites since their increase is considerable during pheochromocytoma and in many situations: asphyxia, hypoglycemia, cold, stress, and physical exercise. Urinary fractional metanephrines are measured after an acid hydrolysis step that allows deconjugation [11].

The abdominal ultrasound is a harmless examination that visualizes the mass, affirms its tissue nature, and studies its impact on the neighboring organs. It also makes it possible to check whether the tumor is localized or multifocal [12]. This examination classically finds an echogenic mass, homogeneous, well limited with clear contours with sometimes calcifications, necrotic or hemorrhagic zones.

Abdominopelvic CT has a major interest in the location of the tumor. It allows a non-aggressive and precise analysis. Its sensitivity is 88% for tumors over 1 cm in diameter [13]. Priority is sought for a periaortic or bladder location. In case of negativity, a spiral thoracic CT scan is carried out, possibly supplemented by imaging of the head and neck. The tumor appears as a homogeneous rounded hyperdense lesion, increasing after injection of the contrast product [12]. Intra-tumor calcifications are present in 10% of cases, and areas of necrosis are much rarer [3].

MRI offers more effective results than CT and can be performed during pregnancy [12]. It shows a T2 weighted sequence hyperintensity with enhancement after gadolinium injection. Marmoluçon [3] in his retrospective series, had assigned him a sensitivity of 100%. However, such a hyper signal can be found in tumors of nervous origin; schwannoma, and ganglioneuroma.

MIBG scintigraphy is a method described in 1981 by Sisson et al. It has been proposed in the topographic diagnosis of pheochromocytoma, in particular those ectopic or of size less than 1 cm. Its sensitivity is 95% and its specificity is around 99%. MIBG has a biochemical structure close to norepinephrine, it is captured and stored by tissues rich in catecholamines. It is ideally marked with iodine 123 of iodine 131 [3].

Positron Emission Tomography (PET) This imaging technique using positron-emitting tracers, the accumulation of which is visualized by a PET camera, has the advantage of being able to detect lesions of the order of 5–10 mm. It has been used with many agents or markers (18 F-fluorodeoxyglucose, 11 C-epinephrine, 18 F-hydroxyphenylalanine …) [14].

The genetic diagnosis must be generalized since the rate of germline mutation can be present between 12 and 24% of cases in patients with a priori a sporadic pheochromocytoma. A systematic genetic study is therefore recommended before any pheochromocytoma, even sporadic in appearance and especially in case of early or multiple formsforms [15].

The treatment of pheochromocytoma is surgical, once the stages of positive and topographic diagnosis have been carried out, the patient must be immediately entrusted to a team of trained surgeons and anesthetists. Surgical excision is conditioned by the precision of the location of the tumor and requires a suitable approach.

The gesture performed is based on the location of the pheochromocytoma, for extra-adrenal tumors and after checking the other chromaffin territories, a simple lumpectomy is performed, the tumor is generally well limited and encapsulated but this excision can extend to the organs neighborhood if they are reached [16].

In the case of vesical pheochromocytoma, cystectomy taking away the whole tumor with an exploration of the ganglionic chains constitutes the therapeutic indication of choice. Transurethral resection does not appear to be reliable. It is likely to be incomplete and seems to generate stronger hypertensive pressures [17]. Some authors insist on the necessity of a wide excision of the tumor with its adipose environment without breaking the capsule to avoid any cell transplant.

The series is short and there is no controlled trial comparing open and endoscopic surgery, or between the two routes of laparoscopic surgery. Subject to sufficient training, endoscopic surgery can be offered to patients with benign pheochromocytoma less than 6 cm in diameter. It allows a lumpectomy and reduces the length of hospital stay while maintaining good safety [18].

It is unpredictable, from a few months to several years and marked by the risk of metastases or recurrences. Late recurrences can occur even when the tumor appears to be benign. In a series of 98 pheochromocytoma operated patients, Van Heerdan had observed six recurrences, three of which were distant, two local and one both local and distant. Local recurrence occurs in the event of incomplete removal of the tumor [19]. The course of recurrences was generally unfavorable in the short term. They have long been accessible to surgery [1].

According to the data in the literature, Chapuis [20] reported an operative mortality of 5.7%. This frequent mortality was favored by the ignorance of the diagnosis of pheochromocytoma is pre-operative. Currently, this mortality is almost zero thanks to advances in medical imaging which allows a pre-operative diagnosis and anesthesia-resuscitation which allows good pre-operative preparation and good control of adrenergic effects during intra-operative [20]. The prognosis remains unpredictable, hence the interest in prolonged monitoring. The rate of survival without recurrence after surgical excision is 75% at 5 years and 45% at 10 years [20]. The median survival is 3 years in the case of metastasis and 4 years in the case of incomplete excision [21].

## 4 Conclusion

Pheochromocytoma is a tumor developed at the expense of chromaffin tissues, adrenal localization represents 90%, extra-adrenal localizations exist, but remain rare (10%). The vesical pheochromocytoma constitutes 13% of ectopic localizations.

The topographic diagnosis is based on CT or MRI. If these are negative, MIBG scintigraphy is a recent method that can be proposed to locate the site of the tumor. These tumors must be diagnosed and treated early to avoid the consequences of adrenergic hypersecretion. The treatment is based on excision surgery (cystectomy for pheochromocytoma of the bladder). Monitoring of patients should be regular and prolonged to detect a possible progression of the disease in the form of metastasis or recurrence.

## Declaration of Competing Interest

The authors report no declarations of interest.

## Funding

We have any funding for your research.

## Ethical approval

The consent to publish this information was obtained from study participants. We confirm that **written** proof of consent to publish study participants are available when requested and at any time.

## Consent

The consent to publish this information was obtained from study participants. We confirm that **written** proof of consent to publish study participants are available when requested and at any time.

## Author’s contribution

Dr. IJ, Dr. AT, Dr. YL Dr. WB analysed and performed the literature research, Pr. MD, Pr. AD, Pr. RA performed the examination and performed the scientific validation of the manuscript. Issam Jandou was the major contributors to the writing of the manuscript. All authors read and approved the manuscript.

## Registration of research studies

Not applicable.

## Guarantor

Issam Jandou.

## Provenance and peer review

Not commissioned, externally peer reviewed.

## Availability of data and material

The datasets in this article are available in the repository of the urology database, Chu Ibn-Rochd Casablanca, upon request, from the corresponding author.
